# Green Food or Greenwashing Trap? The Impact of Greenwashing Perception on Brand Trust and Consumer Food Choices

**DOI:** 10.3390/foods15162877

**Published:** 2026-08-17

**Authors:** Huiyang Zhou, Yun-Chi Tsai, Yong-Yun Cheng, Han-Shen Chen

**Affiliations:** 1 Department of Accounting, Xinjiang University of Finance and Economics, Urumqi 830012, China; zhouhy7659@163.com; 2 Department of Health Industry Technology Management, Chung Shan Medical University, Taichung 40201, Taiwan; vickycai2527@gmail.com; 3 In-Service Master Program of International Health Industry Management, College of Health Care and Management, Chung Shan Medical University, Taichung 40201, Taiwan; fiona263762@gmail.com; 4 Department of Medical Management, Chung Shan Medical University Hospital, Taichung 40201, Taiwan

**Keywords:** consumer perception, food choices, sustainable consumption, consumer behavior, greenwashing perception, brand trust

## Abstract

As green consumption becomes mainstream, deceptive marketing in the food industry poses a significant threat to the sustainability of food systems. This study aimed to investigate how consumers’ psychological decision-making mechanisms and food choices are influenced by corporate greenwashing. This study utilized a quantitative cross-sectional design, integrating environmental cognition, greenwashing perception, and brand trust into an extended Theory of Planned Behavior (TPB) framework. Survey data collected from 445 respondents in Taiwan, using a fictional plant-based food (oat milk) brand as a scenario stimulus, were analyzed using structural equation modeling (SEM). The findings reveal that environmental knowledge and awareness significantly enhance greenwashing perception, which significantly diminishes brand trust, thereby diminishing positive sustainable food choices. Notably, environmental awareness positively moderated the relationship between greenwashing perception and brand trust. Furthermore, the interaction between perceived behavioral control and attitude toward food choices was not significant, demonstrating that emotional rejection substantially outweighs rational evaluation when faced with food-related deception. This study re-examines the rational assumptions of the traditional TPB, revealing that moral defense mechanisms play a central role in consumer food choices. From a practical perspective, the findings urge food enterprises to shift from symbolic green marketing to high transparency, which is essential for restoring brand trust and avoiding consumer boycotts.

## 1. Introduction

In recent years, as the transition toward sustainable diets has accelerated, green consumption has become a mainstream trend in the global food market. However, the pervasive practice of corporate “greenwashing” in the food industry—where enterprises exploit deceptive, vague, or unsubstantiated environmental claims (e.g., “100% natural,” “organic,” or “eco-friendly packaging”)—severely threatens brand equity and sustainable food systems [1,2]. Although previous studies have confirmed that greenwashing significantly diminishes brand trust and behavioral intentions [3,4], there remain critical theoretical gaps. Past research has predominantly emphasized the behavioral outcomes of greenwashing, largely overlooking the psychological mechanisms through which consumers deconstruct these deceptive food labels through “environmental cognition” [5]. Furthermore, most studies focused on Western markets, leaving East Asian contexts—such as Taiwan, which is characterized by high urbanization, a prevalent dining-out culture, extraordinarily high corporate social responsibility (CSR) expectations, and rapid digital information dissemination—underexplored.

Greenwashing in the food sector creates a complex market structure in which “choosing is easy, but identification is difficult”. The covert nature of deceptive food marketing often plunges environmentally conscious consumers into “green confusion” [6], manifesting as a profound “attitude–behavior gap” in sustainable food consumption [7]. To effectively predict consumers’ avoidance or boycott intentions toward greenwashed food products, it is insufficient to approach the issue solely from a knowledge perspective. Therefore, this study employs the Theory of Planned Behavior (TPB) [8] as its foundational framework. We innovatively extend the traditional TPB by integrating “environmental knowledge” and “environmental awareness” as crucial antecedents, examining how “greenwashing perception” attenuates brand trust and reshapes consumers’ attitudes toward food choices.

Crucially, this study addresses the behavioral dilemma of “knowing it is greenwashing but finding it difficult to refuse” in daily dietary decisions [9]. By investigating the moderating roles of environmental awareness and perceived behavioral control (PBC) and utilizing a fictional plant-based food (oat milk) brand as a scenario stimulus, this study specifically aims to examine the empirical mechanisms through which greenwashing perceptions influence consumer brand trust and self-reported behavioral intentions within an extended Theory of Planned Behavior (TPB) framework. Rather than evaluating general psychological decision-making mechanisms or direct real-world dietary choices, this research focuses on how selected constructs—namely perceived greenwashing, brand trust, perceived behavioral control, and subjective norms—interact to shape purchase and boycott intentions toward food products. Ultimately, this study bridges the literature gap regarding the intersection of “identification mechanisms and behavioral theory” in the context of food marketing. Practically, this study provides actionable, empirically grounded insights for policymakers to standardize food eco-labels and for sustainable food brands to communicate authentic environmental values, thereby fostering a highly accountable sustainable food system.

## 2. Literature Review and Hypothesis Development

### 2.1. Greenwashing Marketing and Brand Trust

The core issue of greenwashing in the food industry lies in enterprises strategically exploiting information asymmetry to convey over-embellished, selectively disclosed, or false environmental and health claims. From the perspective of signaling theory, environmental claims (e.g., “organic,” “carbon-neutral,” or “100% natural”) should inherently act as positive signals conveying food quality and corporate responsibility; however, greenwashing behaviors distort these signals into tools that mislead consumers. Although such manipulative tactics may stimulate short-term sales by attracting health- and environment-conscious consumers, they carry significant risks of credit defaults [3,10].

When a gap emerges between actual environmental performance and the projected green image, this integrity deficit triggers a cognitive dissonance. This translates into a “sense of psychological betrayal” and perceived risk, severely eroding brand trust, the core asset that reduces decision-making uncertainty in food consumption [11]. Once trust is destroyed, a “spillover effect” occurs in which consumers harbor skepticism toward all future claims, leading to a long-term collapse in purchase intention [4,6].

Furthermore, because consumers often lack technical expertise when evaluating sustainable food products, deceptive claims easily trigger “green confusion” [5]. Feeling misled, consumers discount the perceived benefits and develop protective negative attitudes. This widespread skepticism damages specific food enterprises and elevates transaction costs across the entire green market [9,11]. In Taiwan, where CSR expectations are exceptionally high, there is minimal tolerance for corporate hypocrisy in the food industry. Greenwashing is not merely a marketing failure but also a profound crisis of brand trust. Higher greenwashing perception enables consumers to detect discrepancies between claims and facts, prompting them to question a food brand’s integrity. This direct link between “perception” and “trust collapse” is key to predicting shifts in consumer food choices and avoidance intentions [4,12]. Therefore, this study proposes the following hypotheses:

**H1.**  *Consumers’ greenwashing perceptions significantly and negatively affect brand trust.*

**H2.**  *Brand trust significantly and negatively affects consumers’ avoidance intentions toward the food brand.*

### 2.2. Environmental Knowledge and Greenwashing Perception

Whether consumers can accurately see through greenwashing traps in a complex food market largely depends on their internal environmental cognitive abilities. These capabilities are not unidimensional and are jointly associated with factual “environmental knowledge” and value-laden “environmental awareness” [13].

From the information processing perspective, environmental knowledge is viewed as an instrumental resource equipped with an “error detection function.” This knowledge encompasses an individual’s scientific understanding of sustainable agriculture, food ecolabelling, and food supply chains. Consumers with specialized environmental knowledge possess stronger analytical skills to benchmark corporate claims against realistic standards, effectively raising critical skepticism toward vague and unsubstantiated green rhetoric on food packaging [3,12]. This knowledge reservoir significantly mitigates the misleading effects of information asymmetry, allowing consumers to exhibit higher error-correcting efficacy when faced with “baseless” or “hidden trade-off” greenwashing messages [14].

However, environmental awareness acts as a “psychological motivational factor” in the perception process of the latter. Environmental awareness reflects an individual’s level of concern for the natural environment and value orientation toward sustainable diets. Consumers who are highly attentive to environmental issues tend to exhibit stronger information-seeking motives: they actively verify the underlying motives and actual environmental performance of food brands rather than passively accepting advertising messages [13]. Studies indicate that when environmental awareness is coupled with environmental knowledge, consumers form a “preventive orientation.” This not only heightens their sensitivity to greenwashing signals but also effectively filters out heuristic fallacies caused by visual imagery (e.g., the illusion created by eco-friendly food packaging or natural imagery), thereby improving their overall perception accuracy [6,14].

In the current consumption environment rife with “green confusion,” environmental cognition serves not only as an antecedent to food choices but also as a self-protective mechanism for individuals who engage in ethical consumption. Consumers with a profound cognitive foundation are more likely to generate a sense of perceive risk when encountering greenwashing behaviors, prompting them to adopt a more prudent evaluation path to avoid falling into food enterprises’ image traps [9,11]. Therefore, elevating consumers’ environmental cognition levels is regarded as a core strategy for countering corporate greenwashing behaviors and safeguarding food-market transparency. Accordingly, this study hypothesizes the following:

**H3a.**  *Environmental knowledge significantly and positively affects consumers’ perceptions of greenwashing.*

**H3b.**  *Environmental awareness significantly and positively affects consumers’ perception of greenwashing.*

### 2.3. Theory of Planned Behavior and Integration of the Core Model

Although the TPB [8] is foundational in environmental behavior research, contexts involving deceptive food marketing and food integrity risks necessitate extending its framework to capture the complex cognitive dynamics of dietary decisions [3]. This study integrates greenwashing perception as a critical antecedent to reshaping traditional TPB constructs. Regarding attitudes, detecting inconsistencies between a food enterprise’s green claims and actual practices triggers “green skepticism” and expectation disconfirmation. This rapidly transforms consumers’ positive environmental intentions into negative brand evaluations, directly diminishing their purchasing attitudes toward greenwashed food products [4,6,15].

For subjective norms, the collapse of brand integrity activates social evaluation pressure. When environmentally conscious groups perceive greenwashing as unethical, normative social conditioning compels individuals to develop avoidance intentions to align with their group’s values [1,12,13]. Furthermore, greenwashing deliberately exploits information asymmetry, inducing “green confusion,” which severely diminishes consumers’ PBC. This elevated interpretative difficulty leaves consumers feeling inadequate and reduces their self-efficacy in making sustainable food choices [5,9]. Such a loss of control is central to explaining the “attitude–behavior gap”: without sufficient confidence to interpret vague food labels, even brands with high environmental awareness struggle to translate it into actual purchases [16,17]. Consequently, these cognitive variables dynamically interact with TPB constructs to drive or inhibit consumers’ food choices during integrity crises. Therefore, this study hypothesizes the following:

**H4a.**  *Consumers’ attitudes significantly and negatively affect their behavioral intentions (i.e., avoidance of greenwashed food).*

**H4b.**  *Consumers’ subjective norms significantly and negatively affect their behavioral intentions.*

**H4c.**  *Consumers’ PBC significantly and negatively affects their behavioral intention.*

### 2.4. Moderating Roles of Perceived Behavioral Control and Environmental Awareness

Although environmental cognition enhances greenwashing identification, a profound “attitude–behavior gap” often impedes actual dietary decision making [7]. To resolve this dilemma, this study posits that “environmental awareness” and “PBC” serve as critical boundary conditions that translate cognition and attitudes into definitive food choices [16].

First, environmental awareness shapes consumers’ tolerance of corporate hypocrisy. For highly aware individuals, identifying food greenwashing triggers severe psychological inconsistency, amplifying the destructive impact of perceived deception on brand trust [3,13]. Conversely, consumers with lower awareness may lack sufficient value identification, thereby weakening their critical evaluation of brand trust when faced with false environmental claims on food packages.

Second, translating a negative attitude into a definitive boycott requires resource feasibility and self-efficacy governed by PBC. Even if consumers strongly resent a greenwashing food brand, their final behavioral shift is constrained by the availability of authentic green food alternatives and their confidence in interpreting complex food labels [16,17]. High PBC enables consumers to confidently execute avoidance behaviors, effectively bridging the gap between negative attitudes and actual boycott intentions [9]. Conversely, excessive “green confusion” diminishes PBC, engendering a sense of powerlessness that inhibits behavioral resistance despite negative attitudes [5,6]. Therefore, this study hypothesizes the following:

**H5.**  *Environmental awareness significantly moderates the relationship between greenwashing perception and brand trust.*

**H6.**  *Consumers’ PBC significantly and positively moderates the relationship between attitude and behavioral intention.*

### 2.5. Negative Responses to Greenwashing and the Taiwanese Context

The consequences of greenwashing in the food industry extend beyond a passive decline in purchase intention: they provoke “moral outrage” and active behavioral retaliation among consumers. When corporate hypocrisy is exposed, consumers feel betrayed. This translates into strong negative brand attitudes and retaliatory actions such as negative word-of-mouth (NWOM) or collective boycotts [5,18]. This transition from psychological aversion to active “value defense” is particularly pronounced in Taiwan.

Characterized by extraordinarily high expectations for CSR, a prevalent dining-out culture, and rapid digital information dissemination, the Taiwanese market exhibits minimal tolerance for deceptive marketing. Food safety and greenwashing scandals have rapidly spread in online communities [4]. Furthermore, Taiwanese consumers are highly susceptible to peer opinions, which are deeply influenced by East Asian cultural dynamics that emphasize the importance of group norms. Once a food brand is labeled as lacking integrity, normative pressure compels individuals to align with collective boycott actions to demonstrate their support for environmental justice [9,13,19]. In this highly connected and CSR-sensitive context, detection of greenwashing triggers intense emotional rejection and negative evaluations of food brands. Therefore, this study hypothesizes the following:

**H7.**  *Consumers’ perception of greenwashing significantly and negatively affects their attitude toward the food brand.*

### 2.6. Research Framework

**Figure 1:**
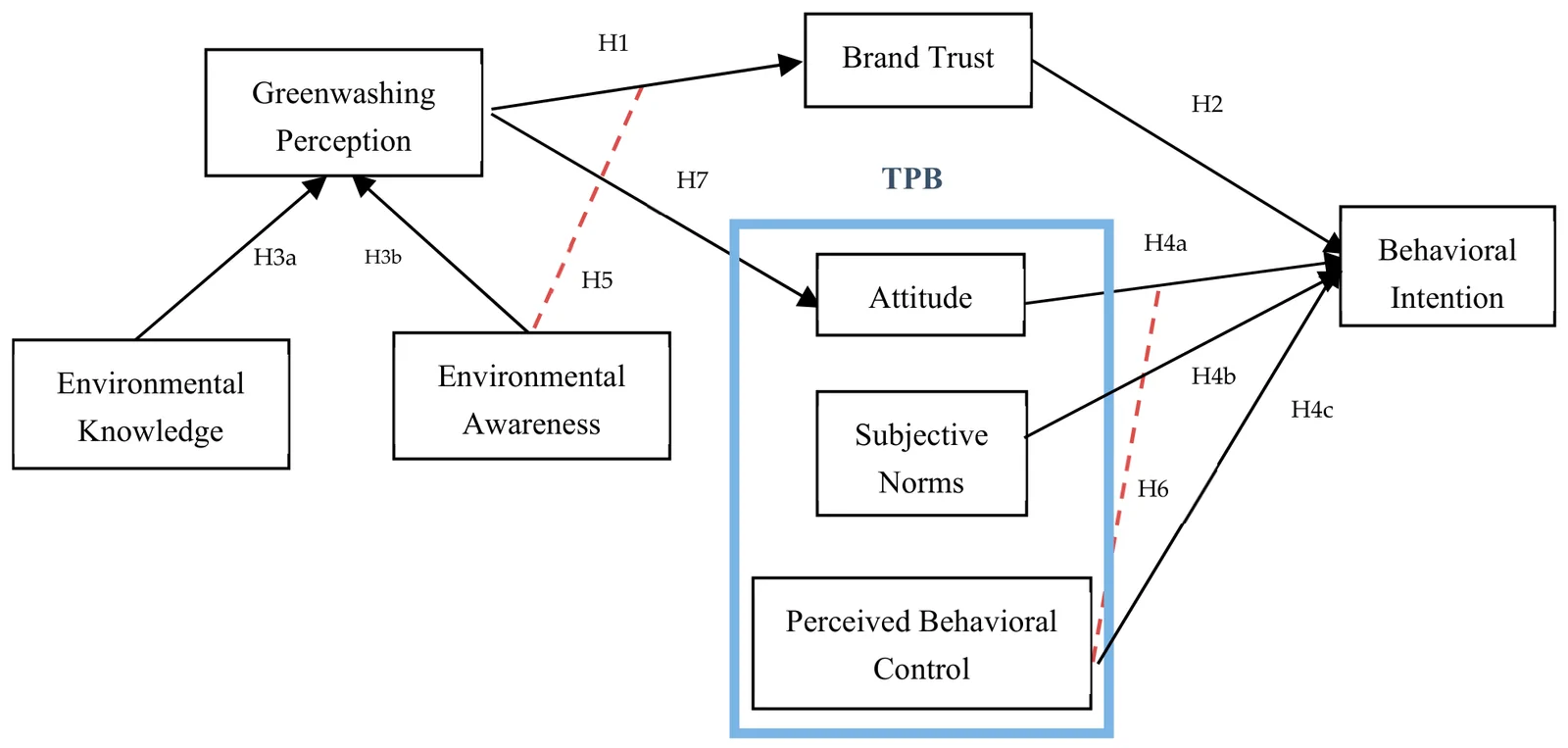
Research framework. Note: Solid lines represent direct pathways, and red dashed lines represent moderating effects (H5 and H6).

Based on the extended TPB framework, this study investigates consumers’ psychological decision-making mechanisms and food choices when they encounter corporate greenwashing in the food industry. The model posits that “environmental knowledge” and “environmental awareness” serve as antecedents driving “greenwashing perception.” This perception subsequently reshapes TPB constructs by directly influencing “brand trust” and “attitude,” ultimately determining “behavioral intention” (which encompasses sustainable food choices, purchase reduction, and brand avoidance). Furthermore, “environmental awareness” and “PBC” were incorporated as moderating variables to clarify the boundary conditions in the attitude–behavior translation process. The conceptual framework is shown in Figure 1.

## 3. Research Methodology

### 3.1. Questionnaire Design

All measurement items were evaluated using a 7-point Likert scale (1 = strongly disagree, 7 = strongly agree) adapted from the literature to ensure content validity in the context of food consumption. Specifically, the measurement instrument comprised 29 items across eight constructs: environmental knowledge (5 items; [20,21]), environmental awareness (4 items; [20,22]), greenwashing perception (3 items; [11]), brand trust (3 items; [23]), attitude (4 items; [21]), subjective norms (4 items; [12,24]), perceived behavioral control (PBC) (3 items; [12,24]), and behavioral intention—operationalized as sustainable food choices and boycott intentions (3 items; [9]).

### 3.2. Data Collection Procedure

#### 3.2.1. Sampling Subjects and Data Collection

A cross-sectional online survey was administered from 17 March to 7 April 2026, targeting Taiwanese consumers aged 18 years and older with independent food purchasing abilities. Utilizing convenience sampling via major social media platforms (e.g., Facebook, Instagram, and LINE), respondents were recruited to voluntarily participate in this anonymous study, which was exempt from formal ethical review according to the National Science and Technology Council guidelines. To ensure data quality, specific screening procedures were applied to eliminate invalid responses (e.g., straight line patterns). Ultimately, 445 valid questionnaires were retained from the 464 submissions. This sample size provides robust statistical power as the respondent-to-item ratio is well above the recommended 10:1 ratio for structural equation modeling (SEM) [25,26].

#### 3.2.2. Experimental Design and Scenario Stimulus

To elicit respondents’ genuine cognitive judgments regarding deceptive food marketing, a “scenario stimulus” was introduced before the formal scale was applied. Respondents were required to read a simulated advertisement for a fictional plant-based food brand (an oat milk brand named “Green Origin”). The exact English translation of the scenario stimulus presented to participants was as follows:

*“Please imagine that you are looking to purchase a more eco-friendly plant-based milk alternative to traditional dairy. While browsing in a supermarket, you are drawn to an oat milk brand named ‘Green Origin.’ The shelf area is decorated with natural straw and green plants, and a sales assistant enthusiastically introduces this annual new arrival: ‘This is our newly developed Pure Earth Series oat milk. The packaging utilizes a special material that resembles recycled paper, prominently featuring a large green eco-logo. We highlight a 100% pollution-free production process and claim that the packaging is fully biodegradable in natural environments. Although there are currently no official eco-labels or international carbon footprint certifications displayed on the package, the brand emphasizes that this is to bypass expensive certification fees and directly pass the savings on to eco-conscious consumers like you.’ However, when you turn to the back of the packaging out of curiosity to inspect the details, you discover that the ingredient list still contains several artificial additives and emulsifiers. Furthermore, fine-print text regarding the ‘fully biodegradable packaging’ specifies that this can only be achieved under ideal conditions in specialized laboratory environments.”*

This experimental setup successfully immersed participants in a realistic food purchasing context and provided a clear and consistent reference for the terms “this food product” and “this brand” in subsequent items, thereby significantly enhancing measurement validity.

### 3.3. Data Analysis Methods

Data analysis was conducted using SPSS Statistics (version 27) and AMOS (version 31) (IBM Corp., Armonk, NY, USA). Descriptive statistics were used to profile respondents’ demographics. Following a rigorous two-step approach, we first evaluated the measurement model’s reliability and validity using Confirmatory Factor Analysis (CFA). Subsequently, SEM with Maximum Likelihood (ML) estimation was employed to test the hypothesized path relationships, moderating effects, and overall model fit. To evaluate the moderating effects (H5 and H6) without inducing high multicollinearity, all continuous predictor and moderator variables were mean-centered prior to constructing the product terms (interaction terms). The Variance Inflation Factor (VIF) values for all resulting terms were confirmed to be below the conservative threshold of 3.3, ensuring the statistical validity of the moderation estimations from SEM.

## 4. Analysis and Results

### 4.1. Demographic Profile

This study collected 464 questionnaires and retained 445 valid responses for analysis after eliminating the invalid samples. The demographic profile revealed a balanced sex distribution (58.7% male and 41.3% female) among consumers. Most respondents were aged between 18 and 54 years, with the 45–54 age group representing the largest segment (27.4%). Regarding educational attainment, 81.6% of respondents held a university degree or higher. Table 1 presents the participants’ detailed sociodemographic characteristics.

### 4.2. Measurement Model: Reliability and Validity

To ensure data integrity and check for potential common method variance (CMV), Harman’s single-factor test was conducted. The results indicated that the first factor accounted for 45.33% of the total variance, falling below the 50% threshold, demonstrating that CMV was not a significant concern [27]. The Kaiser–Meyer–Olkin measure was 0.954, and Bartlett’s test of sphericity was highly significant (*χ*^2^ = 8155.271, *df* = 406, *p* < 0.001), indicating that the data is suitable for factor analysis. Table 2 shows that all standardized factor loadings ranged from 0.710 to 0.893, and the Cronbach’s α and Composite Reliability (CR) values for all constructs were well above the 0.70 threshold, indicating robust internal consistency.

Furthermore, all Average Variance Extracted (AVE) values exceeded 0.50, satisfying the criteria for convergent validity [28,29,30]. Regarding discriminant validity (Table 3), the square root of the AVE value for each construct was greater than its correlation coefficient with all other constructs, fulfilling the Fornell–Larcker criterion.

### 4.3. Structural Model and Hypothesis Testing

This study employed ML estimation to evaluate the structural model and test the hypothesized relationships. The overall model demonstrated a robust fit to the empirical data (χ^2^/df = 1.729, CFI = 0.968, NFI = 0.928, IFI = 0.968, RMSEA = 0.041, and SRMR = 0.032). Although the AGFI (0.889) was marginally below the 0.90 threshold, the excellent performance of the other incremental fit indices confirmed a strong model fit for a complex behavioral model [31].

**Figure 2:**
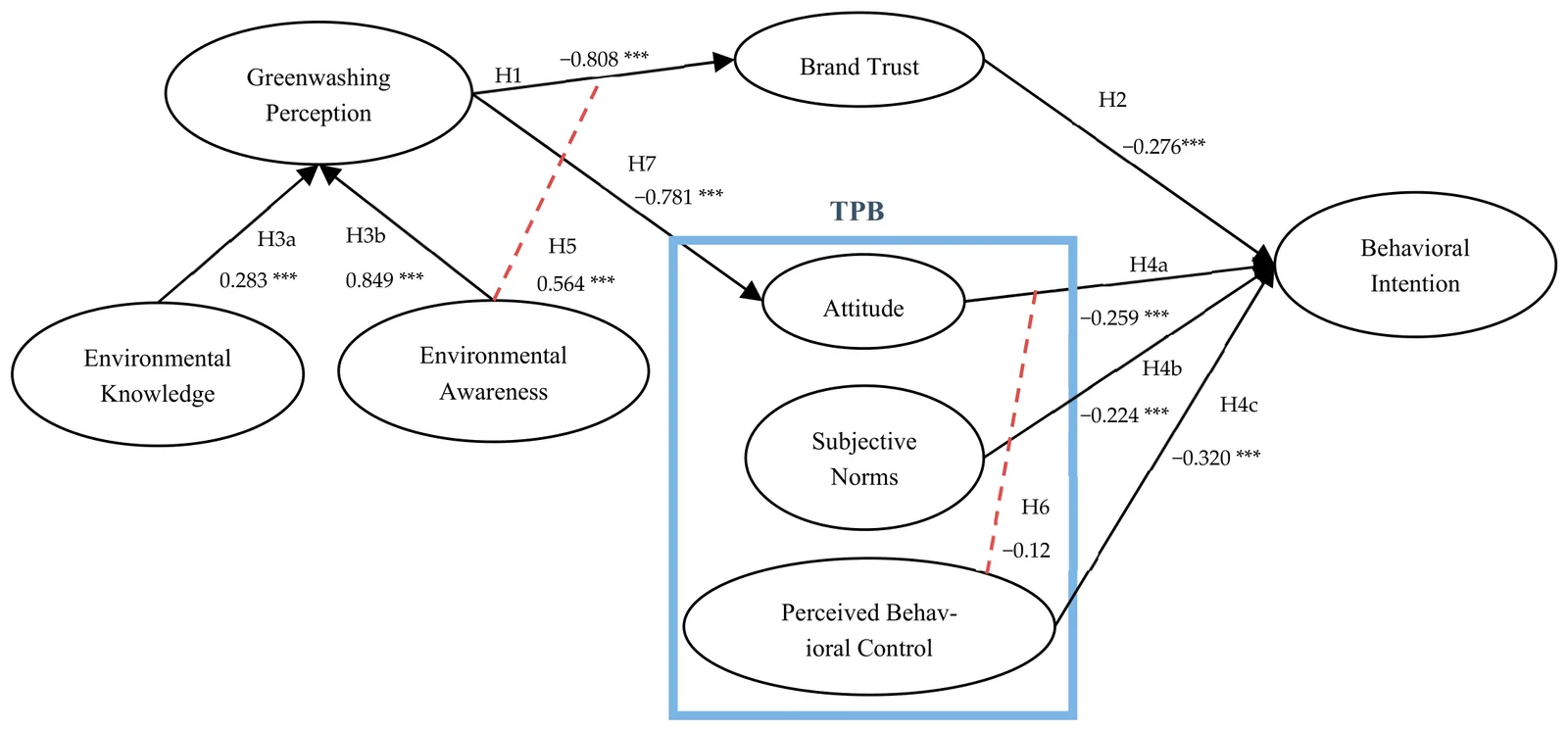
Structural equation model analysis. Note: Solid lines represent direct pathways with standardized coefficients; red dashed lines represent moderating pathways (H5 and H6) with interaction coefficients; *** *p* < 0.001.

The path analysis results, detailed in Table 4 and Figure 2, reveal that greenwashing perception exerted a strong negative impact on brand trust (β = −0.808, *p* < 0.001), which subsequently diminished consumers’ sustainable food choices (β = −0.276, *p* = 0.001), fully supporting H1 and H2. Regarding the antecedents, environmental knowledge (β = 0.283, *p* < 0.001) and environmental awareness (β = 0.849, *p* < 0.001) significantly and positively predicted greenwashing perception, thus supporting H3a and H3b. Within the extended TPB framework, attitude (β = −0.259, *p* = 0.003), subjective norms (β = −0.224, *p* < 0.001), and PBC (β = −0.320, *p* < 0.001) negatively influenced sustainable food choices, supporting H4a, H4b, and H4c.

**Figure 3:**
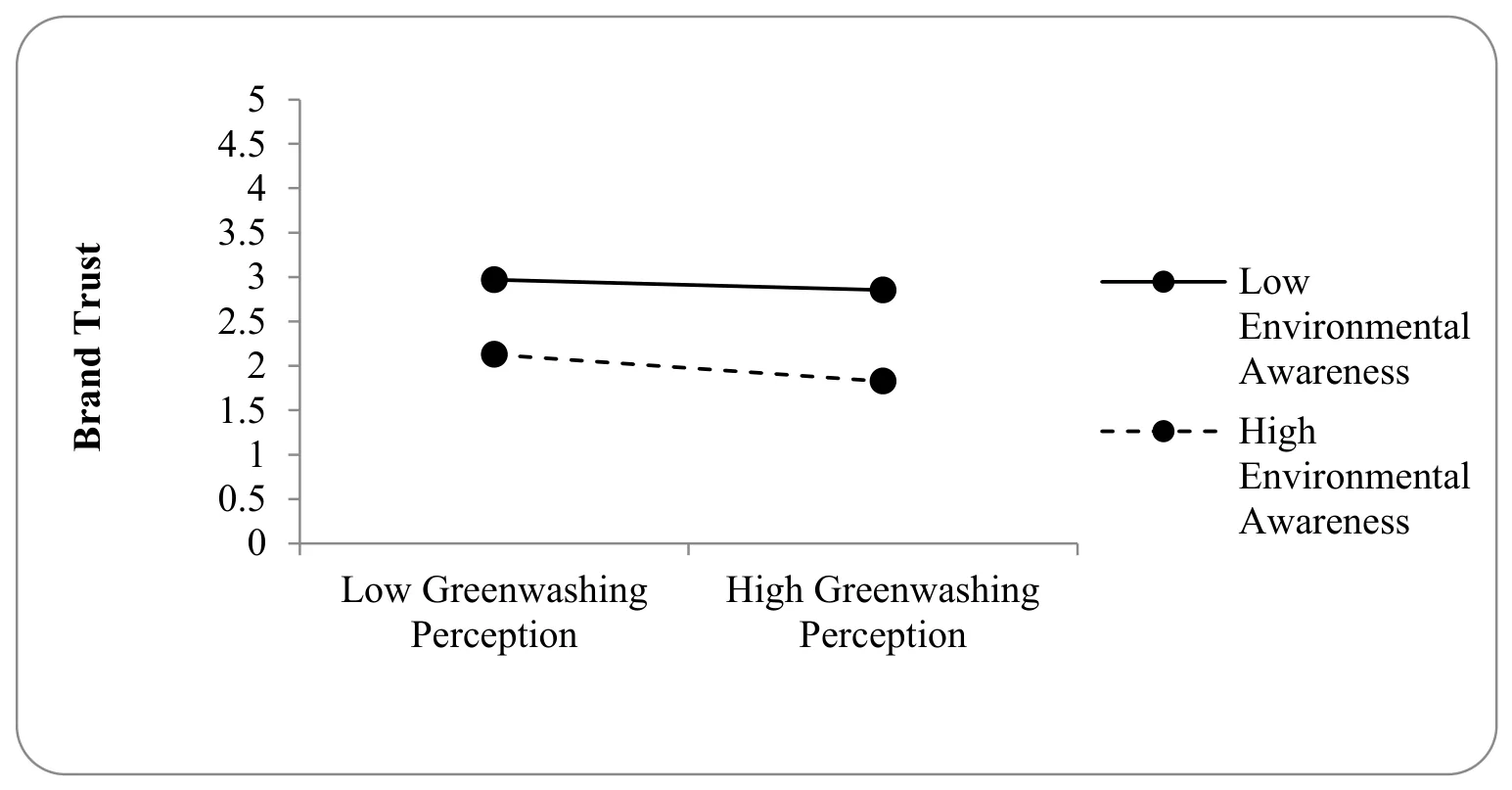
Moderating effect of environmental awareness on the relationship between greenwashing perception and brand trust.

Furthermore, greenwashing perception significantly eroded consumers’ attitudes toward the food brand (β = −0.781, *p* < 0.001), thus supporting H7. Environmental awareness significantly and positively moderated the relationship between greenwashing perception and brand trust (β = 0.564, *p* < 0.001), thus supporting H5. Figure 3 shows that highly aware consumers gave higher trust evaluations, even those with stronger greenwashing perception. Conversely, the interaction between PBC and attitude toward sustainable food choices was not significant (β = −0.12, *p* = 0.793); thus, H6 was not supported.

Figure 3 shows that environmental awareness significantly moderated the negative relationship between greenwashing perception and brand trust, supporting H5. Specifically, for consumers with low environmental awareness, greenwashing perception exerted a sharper, more severe negative impact on brand trust (indicated by a steeper negative slope). Conversely, among consumers with high environmental awareness, the negative effect of greenwashing perception on brand trust was relatively attenuated (indicated by a flatter negative slope). This indicates that higher environmental awareness helps consumers process greenwashing signals more critically or rationally, thereby buffering the drastic collapse of brand trust compared with those with lower awareness.

## 5. Discussion

The results indicate that the perception of greenwashing has a strong and significant negative impact on brand trust (H1 supported). This implies that as consumers become increasingly adept at identifying misleading environmental claims on food packaging, their trust in food brands rapidly declines. Echoing previous studies on deceptive marketing, corporate greenwashing fundamentally attenuates consumers’ confidence in a food brand’s sustainable commitment [23,32]. Detecting false environmental information directly elevates perceived risk, causing consumers to question corporate motives and delivering a severe negative impact to brand trust [11,33]. This trust erosion stems from expectation disconfirmation: when consumers discover a massive gap between symbolic green declarations and substantive environmental actions, long-established brand relationships in the food market deteriorate rapidly [15].

Furthermore, diminished brand trust significantly and negatively affects sustainable food choices, translating to higher avoidance intentions (H2 supported). This indicates that a lack of trust critically hinders sustainable dietary behaviors. In consumer psychology, brand trust plays a foundational role. When deceptive behavior is exposed, previously supportive attitudes become unsustainable, resulting in a significant reduction in positive purchase intentions and driving strong brand avoidance or active boycotts of greenwashed food [3,33,34]. In a food market rife with false information, trust collapse not only severs purchasing pathways but also compels consumers to draw a clear line against deceptive corporations [33].

This study confirms that environmental knowledge and awareness significantly and positively predict greenwashing perceptions (H3a and H3b supported). This result aligns with the “psychological vaccine” perspective in educational interventions, implying that the accumulation of environmental knowledge effectively serves a pre-bunking function, enhancing consumers’ resistance to false green food advertising [35]. Consistent with recent findings on global green consumers, groups with high environmental awareness and knowledge bases possess higher evaluation standards: they demonstrate stronger critical abilities when facing complex food marketing and no longer blindly accept unverified eco-labels [20,36]. This capability mitigates information asymmetry in the food market, acting as an essential psychological defense line for consumer protection [36].

Within the extended TPB framework, attitude, subjective norms, and PBC exerted significant negative impacts on sustainable food choices (H4a, H4b, and H4c supported), with PBC being the strongest inhibitory factor. Specifically, greenwashing perception significantly and negatively affected consumer attitudes toward food brands (H7 supported). This phenomenon is explained by the “green confusion” mechanism: discerning greenwashing triggers strong feelings of deception and confusion, which internalize into a negative attitude and subsequently provoke a strong boycott intention. Cognitive identification is directly linked to the emotional and evaluative rejection of food products [5,9]. Furthermore, Generation Z and younger consumers have extremely high demands for environmental authenticity; detecting symbolic greenwashing drastically shifts their dietary attitudes and rapidly erodes a food brand’s green image [4,13,24].

Regarding the moderating effects, the interaction between environmental awareness and greenwashing perception had a significant positive impact on brand trust (H5). Individuals with high environmental awareness possess complex psychological and defensive mechanisms. These highly concerned consumers are more proactive in perceiving deceptive behavior. Rather than passively abandoning trust, they seek rigorous third-party certifications or use high-transparency information channels (e.g., traceable food supply chains) to re-evaluate and bridge the trust gap [10,36].

Notably, the interaction between PBC and attitude did not have a significant impact on sustainable food choices (H6 unsupported), which presents a critical theoretical nuance that challenges the traditional TPB proposition [8] that perceived control acts as a robust moderator between attitude and intention. In the specific context of food integrity crises, although PBC directly inhibits sustainable food choices, it fails to alter or mitigate the intensity of the negative attitude’s impact on intention.

This phenomenon reflects the profound structural dilemma of the current sustainable food market, which can be explained by the concept of “affective overriding” in behavioral psychology. As described in the “attitude–behavior gap” model [7], when consumers feel betrayed and deceived by a greenwashing food brand, their cognitive evaluation is dominated by moral outrage. Consequently, even if consumers possess high behavioral control, such as abundant knowledge of authentic green food alternatives, extreme convenience in switching brands, or sufficient financial resources or affordable alternatives, these rational, resource-based evaluations cannot effectively neutralize the intense emotional resistance triggered by food greenwashing. In other words, the moral and affective rejection provoked by corporate hypocrisy in the food sector is so intensely unshakable that the traditional moderating transmission mechanism of rational PBC is substantially hindered.

In summary, this study empirically demonstrates that greenwashing fundamentally impairs consumers’ motivation to make sustainable food choices by eroding their trust and transforming their attitudes. From a capital market perspective, exposed greenwashing provokes consumer backlash and generates long-term negative impacts on firm market value [2,19]. Therefore, food enterprises must transition from symbolic packaging to substantive CSR practices. Only by eliminating greenwashing and enhancing information transparency can a robust and sustainable food system be established [37,38].

## 6. Conclusions

### 6.1. Theoretical Contributions

This study advances the literature on sustainable food marketing by integrating greenwashing perceptions and brand trust into the TPB framework. Moving beyond the traditional focus on positive green marketing in the food industry, this study elucidated the negative transmission mechanisms of deceptive environmental food claims. This study offers nuanced empirical insights into the boundary conditions of the TPB, contributing to the literature on sustainable food marketing [8]. While previous applications of the TPB have posited that perceived behavioral control (PBC) effectively moderates the relationship between attitudes and behavioral intentions [7,39], our empirical findings indicate that this moderating effect is not significant in the context of deceptive food greenwashing. Rather than reflecting an absolute emotional override, the non-significance of PBC’s moderating role suggests that when consumers face deceptive greenwashing, the erosion of brand trust plays a dominant role that diminishes the moderating function of subjective control beliefs. Drawing on the moral emotion and consumer ethics literature [40,41], when consumers perceive deceptive greenwashing, the violation of ethical standards evokes intense moral outrage and feelings of psychological betrayal, triggering an “affective overriding” mechanism that neutralizes rational control assessments. This finding indicates that during corporate integrity crises, consumer food choices are governed more by the imperative to avoid deceptive brands than by rational feasibility evaluations.

### 6.2. Practical and Social Implications

From a practical perspective, this study provides vital strategic intelligence for food sector marketing managers. The findings highlight that greenwashing significantly damages brand equity and inflicts real financial damage [19]. To rebuild and maintain green brand trust, enterprises must shift from merely communicating through advertising to making substantive and transparent sustainability commitments. First, food enterprises should leverage continuous monitoring systems to identify potential “green confusion” before it escalates into a brand crisis. Second, food brands should implement high-transparency communication strategies. Beyond traditional certifications, technological solutions often discussed in industry practice—such as blockchain-enabled supply chain tracking or QR-code packaging traceability—represent prospective management options that may help disclose authentic ecological footprints. However, since these specific technologies were not directly measured in our empirical model, they should be viewed as potential strategies that require further empirical verification regarding their efficacy in restoring consumer trust. By enabling consumers to easily verify food origins, brands can reduce information asymmetry and alleviate consumers’ perceived risks regarding their food choices.

From a societal perspective, this study urges policymakers to accelerate the implementation of standardized and strictly regulated food ecolabel systems to penalize deceptive advertising and safeguard market integrity. Integrating greenwashing identification techniques into public environmental and nutritional education can effectively serve as a “psychological vaccine” [35]. By pre-bunking false green claims, educators can cultivate a highly environmentally literate consumer base, which ultimately fosters a robust, healthy, and highly accountable sustainable food system in the long term.

### 6.3. Limitations and Prospects for Future Research

This study has several limitations that provide directions for future studies. First, relying on convenience sampling via online platforms in Taiwan resulted in a sample with a relatively high educational background (81.6% holding a university degree or higher), which may limit the generalizability of our findings. Highly educated individuals typically possess greater baseline environmental knowledge and stronger critical processing capabilities to detect subtle greenwashing cues. Consequently, consumers with lower educational attainment might rely more on simple heuristic cues rather than deep critical processing when evaluating green claims. Future studies are encouraged to employ stratified sampling across more diverse socio-demographic strata to test the boundary conditions of environmental knowledge in detecting greenwashing. Second, while using a fictional brand (“Green Origin”) as a scenario stimulus effectively isolated participants’ preexisting brand attitudes, it inevitably reduced the purchasing realism of the experimental situation. Brand trust in real-world contexts is typically accumulated over long-term interactions. Evaluating trust toward a newly introduced fictional brand may differ from assessing a well-established, familiar brand facing greenwashing allegations, where consumers’ sense of betrayal and psychological resistance could be significantly stronger. Future research could incorporate experimental designs utilizing well-known real-world brands to compare how preexisting brand equity moderates the impact of greenwashing on brand trust. Third, the cross-sectional, self-reported design cannot entirely exclude confounding from the intention–behavior gap in the actual dietary habits of the participants. Respondents evaluating hypothetical cases online might express stronger boycott intentions than they would actually exhibit when shopping in a physical retail store. Furthermore, given the cross-sectional nature of the data, the path relationships in our structural model should be interpreted as structural associations and predictive tendencies rather than strict causal inferences. Future research should employ experimental designs or longitudinal actual food purchase data to evaluate behavioral shifts under various greenwashing typologies (e.g., textual health claims vs. visual eco-friendly packaging). Furthermore, cross-cultural studies incorporating variables such as price sensitivity, sensory attributes, and brand attachment are recommended to comprehensively capture how distinct regulatory environments and cultural dietary norms shape consumer tolerance for “green lies” in the global food market.

## Figures and Tables

**Table 1:** Demographic profile of the respondents (*N* = 445).

Item	Category	Frequency (*N*)	Percentage (%)
Gender	Male	261	58.7%
	Female	184	41.3%
Age	18–24 years old	106	23.8%
	25–34 years old	84	18.9%
	35–44 years old	100	22.5%
	45–54 years old	122	27.4%
	55 years old or older	33	7.3%
Education Level	High school or below	82	18.4%
	College/university	280	62.9%
	Master’s degree or higher	83	18.7%
Monthly Income (NT$)	$20,000 or less (~USD 625)	96	21.6%
	$20,001–$40,000 (~USD 625–1250)	91	20.4%
	$40,001–$60,000 (~USD 1250–1875)	107	24.0%
	$60,001–$80,000 (~USD 1875–2500)	96	21.6%
	$80,001 or more (~USD 2500)	55	12.4%

Note: NT$ = New Taiwan dollar; USD = US dollar. Approximate USD equivalents are provided in parentheses based on an exchange rate of USD 1 ≈ NT$ 32.0.

**Table 2:** Reliability and validity analysis of the Measurement Model.

Construct	Item	Standardized Factor Loading	CR	AVE	Cronbach’s α
Environmental Knowledge	1. The excessive use of chemical fertilizers and pesticides harms the environment.	0.729	0.846	0.523	0.844
	2. Excessive coal burning causes acid rain.	0.745			
	3. Vehicle exhaust emissions affect the air quality.	0.710			
	4. I know how to select products and packaging that reduce the amount of waste that ultimately ends up in landfills.	0.716			
	5. I am knowledgeable about environmental issues.	0.717			
Environmental Awareness	1. I am very concerned about the continuous deterioration of the environmental quality.	0.724	0.844	0.577	0.839
	2. Environmental protection is a significant concern for me.	0.853			
	3. I am enthusiastic about participating in environmental protection issues.	0.710			
	4. Environmental protection will create a better world for my children.	0.742			
Greenwashing Perception	1. I think this product uses misleading words when describing its environmental features.	0.719	0.870	0.692	0.864
	2. I think this product claims to have environmental features, but the claim is vague or seemingly unprovable.	0.893			
	3. I think this product omits or masks important information, making its environmental claims sound better than they actually are.	0.873			
Brand Trust	1. I feel that this brand’s environmental commitment is reliable.	0.784	0.835	0.628	0.828
	2. I feel that this brand’s environmental performance is trustworthy.	0.858			
	3. The brand’s environmental claims are generally trustworthy.	0.731			
Attitude	1. I like the idea of purchasing this brand.	0.824	0.890	0.670	0.888
	2. Purchasing this brand is a good idea.	0.873			
	3. Purchasing this brand is a fashion or trend.	0.744			
	4. I think purchasing this brand is beneficial to me.	0.828			
Subjective Norms	1. Most people who are important to me expect me to purchase this brand for personal use.	0.739	0.846	0.579	0.838
	2. Most people who are important to me think that I should purchase this brand for personal use.	0.729			
	3. People whose opinions I value would prefer me to purchase this brand.	0.771			
	4. Positive evaluations from my friends influenced me to purchase this brand.	0.803			
Perceived Behavioral Control	1. Whether to purchase this brand is entirely up to me.	0.846	0.879	0.708	0.878
	2. I have sufficient resources, time, and opportunities to purchase this brand.	0.833			
	3. I am confident that I can easily purchase this brand if I want to.	0.846			
Behavioral Intention	1. I would feel guilty if I bought a product from this company.	0.743	0.821	0.606	0.817
	2. Whenever possible, I avoid purchasing products from this company.	0.751			
	3. I dislike the idea of owning products from this company.	0.837			

**Table 3:** Correlation matrix and discriminant validity of the measurement model.

Construct	Mean	SD	1	2	3	4	5	6	7	8
1. Environmental Knowledge	6.048	0.808	**0.724**							
2. Environmental Awareness	5.691	0.920	0.596	**0.759**						
3. Greenwashing Perception	5.375	1.255	0.535	0.655	**0.832**					
4. Brand Trust	2.410	0.711	−0.499	−0.715	−0.557	**0.793**				
5. Attitude	2.434	0.978	−0.517	−0.734	−0.553	0.685	**0.819**			
6. Subjective Norms	2.501	0.932	−0.506	−0.639	−0.567	0.524	0.571	**0.761**		
7. Perceived Behavioral Control	2.957	1.207	−0.531	−0.676	−0.543	0.507	0.573	0.512	**0.842**	
8. Behavioral Intention	5.387	1.143	0.591	0.629	0.604	−0.515	−0.554	−0.516	−0.554	**0.778**

Note: The bold values on the diagonal represent the square root of the Average Variance Extracted, and the off-diagonal values are the Pearson correlation coefficients between the constructs.

**Table 4:** Results of path analysis and hypothesis testing.

Hypothesis Path	Unstandardized Coefficient	S.E.	C.R.	*p*	Standardized Coefficient	Conclusion
H1: Greenwashing Perception → Brand Trust	−0.420	0.031	−13.767	<0.001	−0.808	Supported
H2: Brand Trust → Behavioral Intention	−0.440	0.138	−3.180	0.001	−0.276	Supported
H3a: Environmental Knowledge → Greenwashing Perception	0.414	0.071	5.863	<0.001	0.283	Supported
H3b: Environmental Awareness → Greenwashing Perception	1.132	0.087	13.075	<0.001	0.849	Supported
H4a: Attitude → Behavioral Intention	−0.256	0.086	−2.991	0.003	−0.259	Supported
H4b: Subjective Norms → Behavioral Intention	−0.310	0.093	−3.330	<0.001	−0.224	Supported
H4c: Perceived Behavioral Control → Behavioral Intention	−0.241	0.051	−4.747	<0.001	−0.320	Supported
H5: Environmental Awareness × Greenwashing Perception → Brand Trust	0.060	0.006	10.372	<0.001	0.564	Supported
H6: Perceived Behavioral Control × Attitude → Behavioral Intention	−0.012	0.046	−0.262	0.793	−0.12	Unsupported
H7: Greenwashing Perception → Attitude	−0.653	0.047	−13.945	<0.001	−0.781	Supported

## Data Availability

The data supporting the findings of this study are not publicly available because the dataset includes personal data from participants who consented under the conditions of confidentiality and non-disclosure, and we are obliged to uphold privacy rights. However, the data are available from the corresponding author, H.-S.C., upon reasonable request, and with respect to the aforementioned constraints.
